# Molecular Detection and Genotyping of *Cryptosporidium* spp. Isolates from Bats in Colombia

**DOI:** 10.1007/s11686-023-00697-8

**Published:** 2023-08-02

**Authors:** Carlos Ramiro Silva-Ramos, Juliana Noriega, Rafael F. Fajardo, Sandra M. Chala-Quintero, Adriana Del Pilar Pulido-Villamarín, Jairo Pérez-Torres, Rubiela Castañeda-Salazar, Claudia Cuervo

**Affiliations:** 1 Grupo de Enfermedades Infecciosas, Departamento de Microbiología, Facultad de Ciencias, Pontificia Universidad Javeriana, Bogotá, Colombia; 2 Unidad de Investigaciones Agropecuarias (UNIDIA), Departamento de Microbiología, Facultad de Ciencias, Pontificia Universidad Javeriana, Bogotá, Colombia; 3 Unidad de Ecología y Sistemática (UNESIS), Laboratorio de Ecología Funcional, Facultad de Ciencias, Pontificia Universidad Javeriana, Bogotá, Colombia

**Keywords:** Cryptosporidium, Molecular detection, Bats, Colombia

## Abstract

**Purpose:**

Cryptosporidiosis is a zoonotic infectious disease caused by the protozoan parasite *Cryptosporidium* spp., frequently found in several animal species, including bats. Several *Cryptosporidium* genotypes have been described in bats worldwide, suggesting that bats are infected by host-specific *Cryptosporidium* spp. To date, there are no published reports about *Cryptosporidium* spp. in bats from Colombia. Therefore, this study aimed to determine the presence and molecular diversity of *Cryptosporidium* spp. in Colombian bats.

**Methods:**

A total of 63 gut samples from three bat species served for molecular detection of *Cryptosporidium* spp. 18S rDNA gene by qPCR. The sequenced amplicons were used in subsequent phylogenetic analyses to identify them as species or genotypes.

**Results:**

*Cryptosporidium* spp. qPCR detection occurred in 9.5% (6/63) of bat intestines, and four sequences represented two new genotypes, called *Cryptosporidium* bat genotypes XIX and XX, were identified.

**Conclusions:**

This study describes the detection of two novel *Cryptosporidium* bat genotypes, in two species of bats from a region of Colombia, requiring further studies to determine the relationhip between *Cryptosporidium* and bats in Colombia.

## Introduction

Cryptosporidiosis is a zoonotic infectious disease caused by the enteric protozoan *Cryptosporidium* spp., which infects several hosts, including reptiles, birds and mammals [1, 2]. Human cryptosporidiosis can be acquired by direct contact with infected humans or animals, or indirectly by contact with *Cryptosporidium* fecally contaminated material, such as water, food and fomites [3]. Generally, the disease can develop asymptomatically or produce acute diarrhea, although in some cases, *Cryptosporidium* can be an opportunistic pathogen and produce severe forms of the disease that can lead to death to specific populations such as immunocompromised patients [1, 4]. Currently, at least 44 *Cryptosporidium* species have been officially described, and more than 120 genotypes have been identified, which may be formally recognized as species in the future as some *Cryptosporidium* species were initially identified as genotypes [5]. Of all the validated *Cryptosporidium* spp. to date, 29 infect different mammalian species, of which 19 species and four different genotypes have been reported in humans [5].

*Cryptosporidium* spp. have been detected in several domestic and wild animals [2]. Bats (order Chiroptera) represent the most diverse group of mammals; and after humans and rodents, bats are the group with the largest number of individuals worldwide, which can be found on all continents except Antarctica, and inhabit a wide variety of natural (e.g. forests, caves) and human constructions (e.g. abandoned houses, under bridges) [6]. Bats play crucial ecological roles, such as fertilization, pollination, seed dispersal and control of arthropod populations which can act as crop pests or vectors of infectious diseases [7]. Bats are unique among mammals because they are the only known flying mammals and because of their extensive longevity [6]. In addition, the bat's immune system allows them to harbor some of the most pathogenic infectious agents asymptomatically [8, 9].

Several different species of *Cryptosporidium* have been found in bats worldwide, including *Cryptosporidium parvum* and *Cryptosporidium hominis* [10, 11], which are associated with human diseases, increasing the likelihood that bats could be acting as potential carriers and disseminators of *Cryptosporidium* spp. of human health importance [10, 11]. In addition, sequencing of the 18S rDNA gene has facilitated the description of a large number of *Cryptosporidium* genotypes in bats, which were designated with Roman numerals from I to XVIII [12, 13].

To date, 217 species of bats present in Colombia have been officially recognized [14]. According to RELCOM (“Red Latinoamericana y del Caribe para la Conservación de los Murciélagos”), the Macaregua cave, located in the department of Santander, is considered the site with the greatest richness in bat species to date, housing at least ten different species of bats, three of which (*Carollia perspicillata, Mormoops megalophylla* and *Natalus tumidirostris*) inhabit the cave permanently [15, 16]. In Colombia and throughout the world, caves and their surrounding areas are invaded by humans due to spelunking, ecotourism, accelerated urbanization and the increase in agriculture, which encourages and favors the migration of bats to other regions, modifying their roles in the ecosystem processes in which they participate, affecting the well being and benefits that people receive from their ecological functions. On the other hand, when migrating, bats can reach urban areas, increasing the likelihood of direct contact with humans and domestic animals or contaminating environmental sources with their excreta [17].

Although several studies have demonstrated the presence of *Cryptosporidium* spp. in bats globally, no published reports on these parasites are still available from Colombia. Therefore, this study aimed to determine the presence and molecular diversity of *Cryptosporidium* spp. in bats from the Macaregua cave, department of Santander, Colombia, to understand the possible role of bats in the transmission cycle of cryptosporidiosis.

## Materials and Methods

### Bat Samples

During September 2014, June 2015 and October 2018, bats from three different species: *Carollia perspicillata*, *Mormoops megalophylla* and *Natalus tumidirostris*, were randomly captured inside the Macaregua cave, located in the “vereda Las Vueltas”, municipality of Curiti, Department of Santander, Colombia (06 39′36″N; 73 06′32″W, 1,565 m elevation) [15]. Gut samples from each bat were extracted and stored in 70% ethanol at 4 °C at the Molecular Parasitology Laboratory of the Pontificia Universidad Javeriana, before DNA extraction.

Permits for bat capture and sampling were granted by the ‘Ministerio de Ambiente y Desarrollo Sostenible’ and ‘Autoridad Nacional de Licencias Ambientales (ANLA)’, Colombia, license No. 0546, which allows the collection of wildlife specimens of biological diversity for noncommercial scientific research purposes to the Pontificia Universidad Javeriana. Approval for the animal procedures was by the Ethics and Research Committee of the Faculty of Sciences of the Pontificia Universidad Javeriana (ID 5696).

### DNA Extraction

DNA was extracted from 25 mg of bat intestine using the commercial kit “High Pure PCR Template Preparation kit (Roche Diagnostics^®^, Mannheim, Germany)” according to the manufacturer's instructions. After each extraction procedure, to assess the integrity of the DNA and to rule out the presence of PCR inhibitors in the sample, the DNA obtained was quantified using NanoDrop 2000 (Thermo Scientific, Wilmington, DE, United States), and its quality was tested with a conventional PCR targeting a 940 base pair fragment of the cytochrome b (*cyt*B) gene of small mammals using the primers CytB-Uni-F (TCATCMTGATGAAAYTTYGG) and CytB-Uni-R (ACTGGYTGDCCBCCRATTCA) according to the procedure previously described [18]. Subsequently, the amplification products were separated in a 1% agarose gel run through electrophoresis and stained with SYBR^™^ Safe DNA Gel Stain (Invitrogen®, Waltham, MA, USA).

### Detection of *Cryptosporidium *spp. Using a Real-Time PC*R*

The detection of *Cryptosporidium* spp. by qPCR was done using the commercial PowerUp^™^ SYBR^™^ Green Master Mix kit (Applied Biosystems^®^, Austin, TX, USA) according to the manufacturer’s instructions. A mix containing 5 μL 2 × SYBR Green Master Mix, 1 μL of 5 μM primers, 2 μL UltraPure dH2O and 2 μL DNA was used for all PCR reactions. Primers Cr250 (5′-GGAATGAGTKRAGTATAAACCCC-3′) and Cr550 (5′-TGAAGGAGTAAGGAACAACCTCC-3′) [19] served for amplification of a 530 bp fragment of the 18S rDNA gene of *Cryptosporidium* spp. All qPCR reactions were performed using the Applied Biosystems^™^ QuantStudio^™^ 3 Real-Time PCR System (Applied Biosystems^®^, Foster City, CA, USA), using the following program: 50 °C/2 min; 95 °C/2 min; 40 cycles of 95 °C/30 s, 60 °C/45 s and 72 °C/1 min. Melting curve analysis was performed immediately after the last amplification step by heating the samples at 95 °C for 15 s, cooling to 60 °C for 1 min and heating them to 95 °C for 15 s. Bioinformatics analysis of the *18S rDNA* genes for *C. parvum* (GenBank No: S40330.1), *Cryptosporidium muris* (GenBank No: X64342), *Cryptospordium andersoni* (GenBank: AB089285.2) and *Cryptosporidium canis* (GenBank: AF112576) showed that the expected melting temperature (Tm) for the amplified fragments was approximately 73.4 °C. To avoid unspecific amplifications, only samples with cycle threshold (Ct) values ≤ 40 were considered positive. Each qPCR run involved a positive (*Cryptosporidium* spp. DNA) and two negative controls (sterile water). The qPCR-positive samples were re-amplified through conventional PCR, and the amplicons obtained were purified using a Wizard^®^ DNA Clean-Up System kit (Promega, Madison, WI, USA) and then bidirectionally sequenced using a 3500 genetic analyzer (Applied Biosystems^®^, Foster City, CA, USA).

### Phylogenetic Analysis

The sequences obtained were assembled, edited and compared among each other and with *Cryptosporidium* reference sequences available in GenBank after Clustal algorithm alignment. Successfully sequenced positive samples were further analyzed by phylogenetic analysis using the Neighbor-Joining (NJ) method [20], and the evolutionary distances were computed using the Kimura two-parameter method [21] with 1000 bootstrap replicates. All positions containing gaps and missing data were eliminated, and analyses were performed in MEGA software, version 7 [22].

## Results

A total of 80 intestine samples from three bat species stored at the Molecular Parasitology laboratory of the Pontificia Universidad Javeriana were processed for DNA extraction; of which, only 63 (63/80, 78.8%) were positive for *cytB* amplification, and therefore, were used for amplification of a fraction of the 18S rDNA gene of *Cryptosporidium* spp. by qPCR. Six samples (6/63, 9.5%) from two bat species, *C. perspicillata* and *N. tumidirostris*, were positive for *Cryptosporidium* spp. (Table 1) which had Ct values between 23 and 38, and a Tm value of 76.6 °C ± 1.96.

**Table 1:** Frequency of *Cryptosporidium* spp. detection according to bat species captured in the Macaregua cave, Santander, Colombia

Bat species	Number of processed samples (%)	*Cryptosporidium* spp. *18S rRNA* gene positive samples (%)	*Cryptosporidium* genotypes identified
*Carollia perspicillata*	24 (38.1)	4 (16.6)	*Cryptosporidium* bat genotype XIX
*Mormoops megalophylla*	16 (25.4)	0 (0)	None
*Natalus tumidirrostris*	23 (36.5)	2 (8.7)	*Cryptosporidium* bat genotype XX
Total	63 (100)	6 (9.5)	

All 18S rDNA PCR products were sequenced being all of them, but only four retrieved sequences with the adequate quality were further analyzed; these showed an overall identity of 89.3 to 100% among them.

**Fig. 1:**
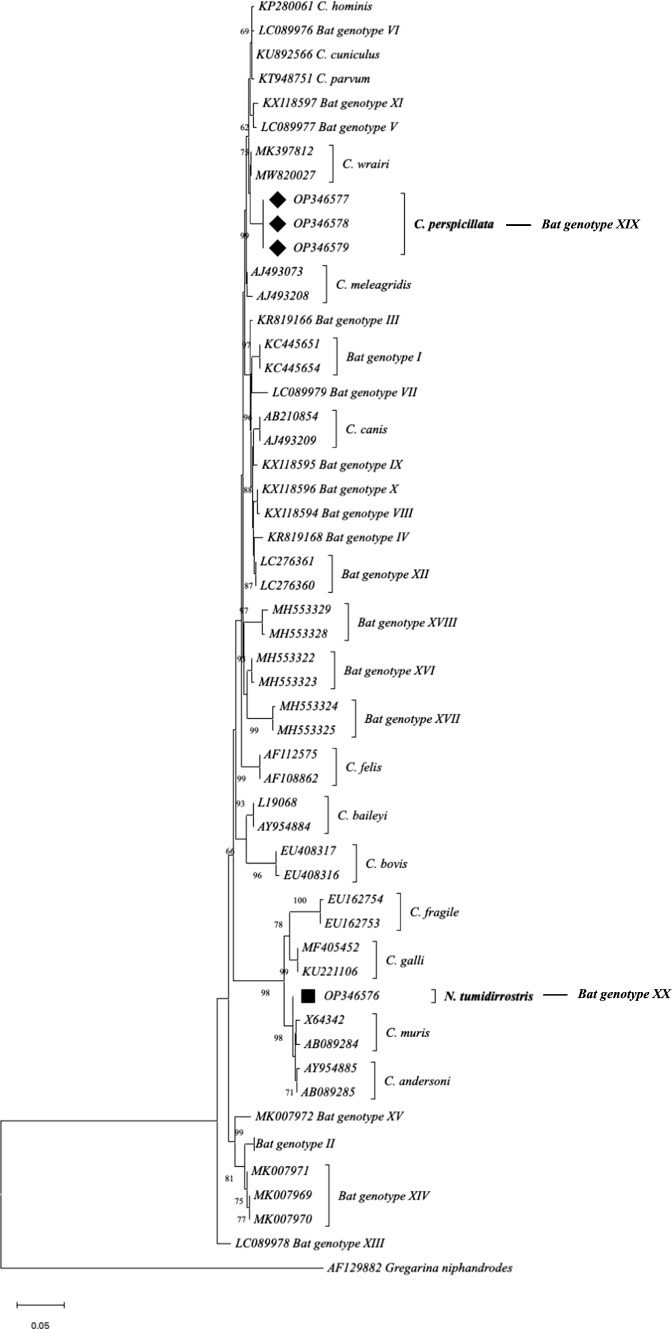
*Cryptosporidium* spp. *18S rDNA* sequence-based phylogenetic tree detected in bats. Sequences retrieved from this study are indicated by symbol: black rhombuses from *C. perspicillata* and black squares from *N. tumidirostris*. The GenBank numbers from the reference sequences are indicated. The *Cryptosporidium* spp. and bat genotypes are listed to the right of each branch (color figure online)

The *Cryptosporidium* spp. NJ analysis of the 18S rDNA gene generated a phylogenetic tree that showed two novel *Cryptosporidium* bat genotypes, namely bat genotype XIX in Seba’s short-tailed bat (*C. perspicillata*) [GenBank accession numbers: OP346577, OP346578, 0P346579] and bat genotype XX in a Trinidadian funnel-eared bat (*N. tumidirostris*) [GenBank accession number: OP346576] (Fig. 1).

Although with low bootstrap values (BP) (62 BP), bat genotype XIX was sister to a clade of *Cryptosporidium wrairi* (Fig. 1), which identity between sequences of bat genotype XIX and *C. wrairi* was 97%. However, bat genotype XX was sister to a clade that included *Cryptosporidium fragile*, *C. galli*, *C. muris* and *C. andersoni* with high bootstrap support (98 BP) (Fig. 1). Bat genotypes from Colombia were not close to other bat genotypes reported from different geographic regions.

## Discussion

Worldwide, only few studies have investigated the presence of *Cryptosporidium* spp. in bats. In the present study, *Cryptosporidium* spp. were detected in bats from a region of Colombia, marking the first report of *Cryptosporidium* in bats from this country and only the second in a Latin American country after a study conducted in Brazil [12]. The frequency of *Cryptosporidium* infection in bats in the present study was 9.5%, which is within the range reported in the scientific literature, where infection frequencies in bats range from 2.1% [10] to 16.3% [12].

Four of the six positive samples for *Cryptosporidium* spp. were successfully sequenced. These sequences clustered in two different genotypic groups, designated as *Cryptosporidium* bat genotypes XIX and XX. Three of these sequences were from *C. perspicillata*, which clustered as an independent clade with the closest proximity to *C. wrairi*, a species described from laboratory guinea pigs and other rodent species that can infect humans but without evidence of disease [23–25]; and one sequence was from *N. tumidirostris*, which formed a clade with two *Cryptosporidium* spp.: *C. muris*, a species described in *Mus musculus* (domestic mouse) and *C. andersoni*, a species previously described in *Bos taurus* (domestic cattle) [26, 27]; both of them zoonotic species which can infect humans and several animal species [28–31].

To date, 18 *Cryptosporidium* bat genotypes have been identified among 18 different bat species captured in China [13], United States [10], Czech Republic [10], Philippines [32, 33], Australia [11], Japan [34], Nigeria [33], and Brazil [12] (Table 2). Although most of the *Cryptosporidium* bat genotypes have been detected in specific bat species, some of them such as genotypes I, XIII and XVI, have been detected in more than one bat species (e.g. genotype I in *Rhinolopus sinicus* and *Aselliscus stoliczkanus*; genotype XIII in *Hipposideros fulvus* and *Rousettus leschenaultia*; and genotype XVI in *Artibeus planirostris*, *Artibeus lituratus* and *Platyrrhinus lineatus*) [12, 13], suggesting that apparently *Cryptosporidium* bat genotypes may not be host specific. Given the limited research on *Cryptosporidium* among bats worldwide, it is likely that several novels and undescribed *Cryptosporidium* genotypes are circulating among different bat species in many regions throughout the world, especially considering that the order Chiroptera represents the second most diverse order of living animals [35]. Furthermore, detecting different *Cryptosporidium* genotypes from bats of the same geographical region would suggest that these mammals may be playing a role in the diversification of *Cryptosporidium* spp., similar to what happens to other microorganisms such as *Trypanosoma cruzi* and *Bartonella* spp. [36, 37].

**Table 2:** Detection of *Cryptosporidium* species and bat genotypes worldwide

Year—author	Sample proccessed	Country of origin of samples	*Cryptosporidium* species identified	*Crytosporidium* bat genotypes identified	Infection frequency (%)	References
2013—Wang *et al*.	Intestines	China	*Cryptosporidium* spp.	I, II	19/247 (7.7)	[13]
2015—Kváč *et al*.	Feces	Czech Republic and United States	*Cryptosporidium parvum*	III, IV	6/281 (2.1)	[10]
2016—Murakoshi *et al*.	Intestines	Phillipines	*Cryptosporidium* spp.	II, V, VI, VII	4/45 (8.9)	[32]
2016—Schiller *et al*.	Feces	Australia	*Cryptosporidium hominis*	VIII, IX, X, XI	9/281 (3.2)	[11]
2018—Murakoshi *et al*.	Intestines	Japan	*Cryptosporidium* spp.	XII	2/3 (66.7)	[34]
2018—Li *et al*.	Feces	Nigeria	*Cryptosporidium* spp.	XIV, XV	6/109 (5.5)	[33]
2019—Batista *et al*.	Feces	Brazil	*Cryptosporidium* spp.	XVI, XVII, XVIII	23/141 (16.3)	[12]

Interestingly, two *Cryptosporidium* species of medical importance, *C. parvum* and *C. hominis*, have also been detected in bats [10, 11], suggesting that bats may play a role in the eco-epidemiology of cryptosporidiosis [38, 39]. However, the link between bats and *Cryptosporidium* remains poorly understood, and further research is needed to better comprehend this relationship, not only from a medical and veterinary perspective, but also from a biological and ecological perspective since several *Cryptosporidium* genotypes have been continuouslly reported from bats worldwide in the scientific literature. This research is crucial to better understand the transmission dynamics and the range of host species that *Cryptosporidium* can infect, as well as to expand the knowledge about the taxonomy of this parasitic genus since some *Cryptosporidium* species were initially identified as genotypes prior to their formal description as validated species [5]

Although this is the first study in which the presence of *Cryptosporidium* bat genotypes has been reported in Colombia, the study has some limitations related to the low number of samples processed, the use of a single locus for the genotyping of isolates obtained from bats, and the limited geographical representativeness, therefore, it is necessary to carry out more studies to improve the comprehension of the biology, ecology and epidemiology of bat *Cryptosporidium* isolates in Colombia.

## Conclusion

This study describes the first detection and molecular identification of *Cryptosporidium* spp. in bats in Colombia. It also detected the presence of two new *Cryptosporidium* bat genotypes, designated as *Cryptosporidium* bat genotype XIX and XX. These data point to the need for further studies to determine whether bats play a role in *Cryptosporidium* diversification, to identify whether these bat genotypes are novel *Cryptosporidium* species not yet described, and if so, to determine what is the impact of these not yet validated *Cryptosporidium* isolates for human and animal health.

## Data Availability

Raw data generated from this study is available upon personal request from the corresponding author.
